# Supplementary material for: Fear of positive evaluation in borderline personality disorder
Source: PLoS One. 2020 Aug 20;15(8):e0237944. doi: 10.1371/journal.pone.0237944 (PMC7444514; doi:10.1371/journal.pone.0237944)
Supplement: Attachment — Submitted filename: Response to Reviewers.docx [file pone.0237944.s002.docx]

Thank you for the opportunity to revise our manuscript (PONE-D-20-12363) entitled “Fear of positive evaluation in borderline personality disorder”.

We appreciate the feedback on the initial version, and have done our best to address all of the comments/suggestions provided.

We addressed the issues raised by the reviewers as follows:

Reviewer #1: This manuscript examines the role of fear of positive appraisal and rejection sensitivity in borderline personality disorder (BPD) comparing three groups: healthy controls, patients with BPD, and patients with social anxiety disorder. While this paper has the potential to be a contribution to the growing literature about the role of difficulty with positive appraisal and BPD, in its current form is underdeveloped in its literature review as well as its explanation of findings.

1. My first major criticism of this draft is that the introduction suggests that there are distinct features of BPD samples that would suggest fear of positive appraisal, but the findings and implications of these studies could be better explained. This literature is summarized by the authors using 4 papers which are swiftly described in jargon laden terms which makes it difficult to know the actual studies or the relevant take home points. The fifth study, reference 13 is cited to suggest there is a "positivity impairment" in BPD, based on a chapter on social anxiety disorder. More can be clarified about what this positivity impairment involves, and ignores literature of BPD and positive affects and cognition (e.g. Reed & Zanarini, 2011; Reed, Fitzmaurice, and Zanarini 2012). There is no discussion of the existing literature on positive affect and BPD, which is confusing given the literature background presented is sparse and brief. The authors then turn to the role of fear of positive evaluation in social anxiety, using an evolutionary model, but do not elaborate in any way how this would relate to BPD. This paper would be a better contribution if authors gave more space for summarizing more clearly this interesting literature.

**We agree and thank the reviewer for the relevant references. To address the issue, we included a more comprehensive chapter on positivity impairments in BPD in the introduction (P3-4, L46-64): *“****Positivity impairments can be defined as alterations in the experience of positive affect as well as alterations in the processing of positive information (1-3). Concerning the experience of positive affect, there is robust evidence that individuals with BPD experience less positive affect in their daily life (e.g., 4, 5) and report to down-regulate positive affect (e.g., 6). Moreover, individuals with BPD experienced positive affective states (e.g. to feel accepted, to feel safe) and cognitive states (e.g., “I trust myself”) less frequently than individuals with another personality disorder (7). Importantly, these impairments seem to predict recovery of BPD over time (8). Concerning the processing of positive information, research revealed that individuals with BPD process positive information in a more negative manner. For example, individuals with BPD experienced less positive emotions such as pride and happiness after reading self-relevant appreciating sentences (9) and rated positive, self-relevant words as more negative than a non-clinical control group (10). Moreover, individuals with BPD seem to be impaired in the processing of positive social feedback. An experimental study indicated that individuals with BPD integrate negative self-relevant social feedback to a greater extent than positive social feedback (11), while healthy individuals show the opposite updating bias (integrating positive feedback to a greater extent than negative). Another experimental study indicated that individuals with BPD change their social expectations in response to negative, but not positive, social feedback (12). […]“*

2. My second criticism, is that the study hypotheses are nonspecific in terms of what was to be found in the comparison between social anxiety and BPD.

**We did not include a specific hypothesis for the comparison between the two clinical groups, because fear of positive evaluation has not been studied in individuals with BPD before. Theoretical considerations as well as a study in a non-clinical sample by Rodman et al. (2008) point towards high fear of positive evaluation in individuals with BPD. However, to the best of our knowledge, we were not able to formulate a specific hypothesis regarding the level of fear of positive evaluation in individuals with BPD compared to individuals with SAD.**

**In the revised version, we stated our assumptions for the association between social anxiety and rejection sensitivity in BPD more clearly (P6, L105-108; see also reviewer 2, comment 3):** *“Based on theoretical considerations (13), we assumed that specifically in individuals with BPD rejection sensitivity is associated with fear of positive evaluation over and above social anxiety.”*

3. The finding that rejection sensitivity and fear of positive appraisal did not differentiate the groups could be framed in a more specific way and is not clearly explained. Given the lack of overlap between the SAD and BPD groups it is hard to determine how social anxiety drives fear of positive evaluation for the entire sample or how it washes out the effects of rejection sensitivity. The authors do not explain this at all in the conclusions and only reference this study's similarity to a report in an unpublished dissertation. A fuller exploration for why occurred would help the manuscript.

**Thank you for mentioning this. We included a detailed discussion on P17-18, L315-336 (see also reviewer 2, comment 3 and 4).** *“However, the regression analysis revealed that the association between rejection sensitivity and fear of positive evaluation disappeared once controlling for social anxiety and that social anxiety accounts for most of the variance in fear of positive evaluation. Hence, social anxiety might have driven the association between rejection sensitivity and fear of positive evaluation in our sample. […].*

*However, this finding is limited by the fact that social anxiety and fear of positive evaluation were highly correlated in our BPD sample (r = 0.80), which raises the question if the applied questionnaires measure the same underlying construct. Indeed, there is an overlap in some items of both applied questionnaires (e.g., SPIN: “I am afraid of people in authority”, “I avoid activities in which I am the center of attention.”; FPES: “I feel uneasy when I receive praise from authority figures.”, “I don’t like to be noticed when I am in public places, even if I feel as though I am being admired.”). However, the high correlation between both questionnaires was specific for the BPD sample. In healthy participants and the SAD sample, the correlation was lower (r = 0.55, r = 0.44) and comparable to previous studies (14, 15). Moreover, fear of positive evaluation relates to being afraid of others’ positive appraisal, while social anxiety is characterized by fear of negative evaluation and less well researched fear of positive evaluation (e.g., 14, 16). This favors the distinctiveness of both constructs.*

*It is noteworthy that in the BPD sample, different self-report measures were highly correlated. It is possible that that a generalized negative affectivity or a negativity bias (17-20) drives this correlation pattern.”*

4. My last criticism is that the clinical implication of these findings are mentioned but not explicit what would be done clinically in terms of intervention or management. The authors say a therapist should “apply suitable interventions” after exploring the emotional reaction of their patient. Could they specify some examples of suitable interventions?

**We agree that the proposal of specific interventions would improve our article and included suggestions for specific interventions in the discussion (P16, L300-310; see also reviewer 2, comment 5):** *“What are the clinical implications of high fear of positive evaluation in BPD? Therapists should be aware that complimenting or giving positive feedback might be frightening and*/ or *difficult to accept for patients with BPD. Therefore, it could be helpful if the therapist prefaces compliments to the patient by pointing out that this might trigger aversive emotions. Moreover, therapist and patient should explore why a compliment triggers negative emotions (e.g., the compliment is schema-incongruent, the patient is suspicious regarding the intentions behind the compliment). This way, the patient might learn to understand the experience of negative emotions before or after receiving a positive social feedback. In the long-term and accompanied by further interventions (e.g., development of a suitable skill to accept positive feedback), this might help patients with BPD to experience less negative and more positive emotions in the context of a positive social feedback.”*

5. A number of copy editing problems are as follows: Pg. 4, second full sentence, should read “adaptive”, instead of “adaptable”; Pg. 12 last paragraph, should read “explained”, instead of “exlained”, line 58 says letter instead of latter) was. **Thank you, we corrected these errors and have edited the manuscript carefully.**
6. Line 151 – they mention a measure (LPS-4) that isn’t spelled out nor described in their measures. Seems to be a measure of IQ though, so not a main outcome measure, not sure if it needs more description. **The LPS-4 score was used to match groups on IQ, which was exclusively important for the EEG assessment (reported in Weinbrecht et al., 2018). We decided to remove the LPS-4 measure from the manuscript to avoid confusion.**

Reviewer #2: This study investigates fear of positive evaluation in BPD patients as compared to SAD and HC. It’s a well-structured, well-written paper and I only have some minor comments and suggestions for additional discussion.

1. In the intro, the authors seem to make the argument that fear of positive evaluation in BPD patients may have a different nature than in SAD and be more determined by BPD patient’s fear of rejection, following Linehan’s theory. One would assume the authors would expect that rejection sensitivity might have additional explanatory value beyond social anxiety for BPD patients, based upon their intro (and their statement that they base their hypothesis on Linehan’s theory). I wonder why they haven’t put this hypothesis.

**Indeed, we did expect that rejection sensitivity have additional explanatory value beyond social anxiety in BPD patients. However, this hypothesis was based exclusively on theoretical considerations and there was no related empirical evidence available. Therefore, we were hesitant to present a specific hypothesis. We acknowledge that we should have stated our expectations more clearly and changed the relevant passage in “research questions and hypotheses” (P6, L105-108):** *“2) […]. Based on theoretical considerations (13), we assumed that specifically in individuals with BPD rejection sensitivity is associated with fear of positive evaluation over and above social anxiety.”*

2. Some more information may be provided on the nature of the SAD sample and whether there are indications for comorbidity with PDs in the SAD sample. On the other hand, some information on the presence of SAD and other anxiety disorders in the BPD sample. This may help to clarify how different both samples are and what kind of SAD vs BPD sample these are.

**We added further diagnostic information to Table 1 (P6-7, L120-122).**

|  | HC  (*n* = 35) | SAD  (*n* = 29) | BPD  (*n* = 36) |
| --- | --- | --- | --- |
| Female, *n* (%) | 29 (83) | 23 (79) | 31 (86) |
| Age, *M (SD)* | 27.69 (5.66) | 28.83 (6.10) | 28.08 (4.95) |
| Number of comorbid diagnoses, *M (SD)* | 0 | 1.21 (1.15) | 1.70 (1.10) |
| Antidepressant medication, *n* (%) | 0 | 8 (27.59) | 13 (36.11) |
| MDE current, *n* (%) | 0 | 7 (24.18) | 2 (5.56) |
| MDE lifetime, *n* (%) | 0 | 8 (27.59) | 15 (41.67) |
| SAD, *n* (%) | 0 | 29 (100) | 1 (2.78) |
| Other anxiety disorders, *n* (%) | 0 | 0 | 11 (30.56) |
| PTSD, *n* (%) | 0 | 5 (17.24) | 11 (30.56) |
| BPD, *n* (%) | 0 | 0 | 36 (100) |
| AVPD, *n* (%) | 0 | 7 (24.18) | 0 |
| Other personality disorders, *n* (%) | 0 | 1 (3.45) | 3 (8.33) |

HC = Healthy Controls, SAD = Social Anxiety Disorder, BPD = Borderline Personality Disorder; MDE = Major Depressive Episode, PTSD = Posttraumatic Stress Disorder, AVPD = Avoidant Personality Disorder.

3. There is a very high correlation in the BPD sample between social anxiety and fear of positive evaluation of .80. At least in this sample measures of social anxiety and fear of positive evaluation seem to be measuring almost the same construct. I think this needs extra attention from the authors as this may reflect a problem with their measures.

**We completely agree and included a more detailed discussion** **on P17, L322-333 (see also reviewer 1, comment 3)**: *“However, this finding is limited by the fact that social anxiety and fear of positive evaluation were highly correlated in our BPD sample (r = 0.80), which raises the question if the applied questionnaires measure the same underlying construct. Indeed, there is an overlap in some items of both applied questionnaires (e.g., SPIN: “I am afraid of people in authority”, “I avoid activities in which I am the center of attention.”; FPES: “I feel uneasy when I receive praise from authority figures.”, “I don’t like to be noticed when I am in public places, even if I feel as though I am being admired.”). However*, *the high correlation between both questionnaires was specific for the BPD sample. In healthy participants and the SAD sample, the correlation was lower (r = 0.55, r = 0.44) and comparable to previous studies (14, 15). Moreover, fear of positive evaluation relates to being afraid of others’ positive appraisal, while social anxiety is characterized by fear of negative evaluation and less well researched fear of positive evaluation (e.g., 14, 16). This favors the distinctiveness of both constructs. […]”*

4. Remarkably in the BPD sample is the general rather high level of correlations between all variables and fear of positive evaluations (and maybe also among each other) as compared to the other samples showing a more diverse pattern of correlations. Could the authors discuss this difference? Could it be related to a generalized negative affectivity that characterizes BPD?

**Thank you for mentioning this interesting issue. We now discuss the correlation pattern and its potential relation to generalized negative affectivity on P18, L334-336:** *“It is noteworthy that in the BPD sample, different self-report measures were highly correlated. It is possible that a generalized negative affectivity or a negativity bias (17-20) drives this correlation pattern.”*

5. The authors discuss very briefly implications and I would like them to add a bit to this discussion given the prominent view in many (behavioral) approaches to reinforce patients by making compliments and to assist parents in complimenting their (BPD) kids. These findings seem to suggest that such an approach only triggers negative feelings in BPD patients. How do these findings fit with therapeutic recommendations?

**We rewrote the section on clinical interventions** **(P16, L300-310; see also reviewer 1, comment 4). We didn`t want to imply that therapists should withhold positive feedback, but that it might be helpful to be especially attentive when doing so. We tried to clarify this issue:** “*What are the clinical implications of high fear of positive evaluation in BPD? Therapists should be aware that complimenting or giving positive feedback might be frightening and /or difficult to accept for patients with BPD. Therefore, it could be helpful if the therapist prefaces compliments to the patient by pointing out that this might trigger aversive emotions. Moreover, therapist and patient should explore why a compliment triggers negative emotions (e.g., the compliment is schema-incongruent, the patient is suspicious regarding the intentions behind the compliment). This way, the patient might learn to understand the experience of negative emotions before or after receiving a positive social feedback. In the long-term and accompanied by further interventions (e.g., development of a suitable skill to accept positive feedback), this might help patients with BPD to experience less negative and more positive emotions in the context of a positive social feedback.”*

6. The authors may discuss their findings also in relation to other possible explanations in the literature with regard to fear for positive evaluation in BPD patients, that may be targeted in future studies, e.g. problems in social cognition making BPD patients suspicious regarding the intentions behind positive feedback; or the discrepancy between positive evaluations and their own low self-esteem.

**We included a more comprehensive discussion of our explorative item analysis on P15-P16, L285-291**. *“To shed more light on high fear of positive evaluation in BPD, we compared differences between clinical groups on the item level of FPES in an exploratory analysis. Individuals with BPD reported to be more uncomfortable when receiving a compliment than individuals with SAD. It could be speculated that a compliment is incongruent with the negative self concept (e.g., 22) of individuals with BPD. Therefore, a compliment might trigger unwanted negative emotions such as anger (e.g., “the therapist is lying to me”) or shame (e.g., “the therapist has no idea how unworthy I am”).“*

**Moreover, we added aspects that should be targeted in future research on P18, L342-346. We thank the reviewer for the suggestions, which we incorporated into the manuscript.** *“Future studies need to clarify the underlying mechanism of high fear of positive evaluation in BPD. As speculated above, a positive evaluation might be incongruent with the negative self concept (e.g., 22) of individuals with BPD or related to impairments in social cognition in BPD (e.g., 17, 23), which lead to being suspicious regarding the intentions behind a positive evaluation.”*

**We updated the reference list accordingly.**
